# Awareness of Clenching and Underweight are Risk Factors for Onset of Crowding in Young Adults: A Prospective 3-Year Cohort Study

**DOI:** 10.3390/ijerph16050690

**Published:** 2019-02-26

**Authors:** Naoki Toyama, Daisuke Ekuni, Ayano Taniguchi-Tabata, Kota Kataoka, Mayu Yamane-Takeuchi, Kohei Fujimori, Terumasa Kobayashi, Daiki Fukuhara, Koichiro Irie, Tetsuji Azuma, Yoshiaki Iwasaki, Manabu Morita

**Affiliations:** 1 Department of Preventive Dentistry, Okayama University Graduate School of Medicine, Dentistry and Pharmaceutical Sciences, 2-5-1 Shikata-cho, Kita-ku, Okayama 700-8558, Japan; pu171qxi@s.okayama-u.ac.jp (N.T.); de19026@s.okayama-u.ac.jp (A.T.-T.); de18017@s.okayama-u.ac.jp (K.K.); de18053@s.okayama-u.ac.jp (M.Y.-T.); kfujimori@s.okayama-u.ac.jp (K.F.); de421015@s.okayama-u.ac.jp (T.K.); de20041@s.okayama-u.ac.jp (D.F.); mmorita@md.okayama-u.ac.jp (M.M.); 2 Department of Microbiology and Immunology, Columbia University Medical Center, 650 West 168 Street 141 Black Building, New York, NY 10032, USA; coichiro@md.okayama-u.ac.jp; 3 Department of Community Oral Health, Asahi University School of Dentistry, 1851-1 Hozumi, Mizuho, Gifu 501-0296, Japan; tetsuji@dent.asahi-u.ac.jp; 4 Health Service Center, Okayama University, Okayama 700-8530, Japan; yiwasaki@okayama-u.ac.jp

**Keywords:** bruxism, cohort study, malocclusion, underweight, young adults

## Abstract

Bruxism is a parafunctional activity that can seriously affect quality of life. Although bruxism induces many problems in the oral and maxillofacial area, whether it contributes to the onset of malocclusion remains unclear. The purpose of this prospective cohort study was to investigate the association between the onset of malocclusion and awareness of clenching during the daytime in young adults. Among 1,092 Okayama University students who underwent normal occlusion at baseline, we analysed 238 who had undergone a dental examination and had complete data after 3 years (2013–2016). We also performed subgroup analysis to focus on the association between awake bruxism and the onset of crowding (n = 216). Odds ratios (ORs) were calculated using multivariate logistic regression analyses. The incidences of malocclusion and crowding were 53.8% and 44.5%, respectively. In multivariate logistic regression, awareness of clenching was a risk factor for crowding (OR: 3.63; 95% confidence interval [CI]: 1.08–12.17). Moreover, underweight (body mass index < 18.5 kg/m^2^) was related to the onset of malocclusion (OR: 2.34; 95%CI: 1.11–4.92) and crowding (OR: 2.52, 95%CI: 1.25–5.76). These results suggest that awareness of clenching during the daytime and underweight are risk factors for the onset of crowding in young adults.

## 1. Introduction

Bruxism is a parafunctional activity. Although definitions vary [1,2,3,4,5], it is divided into two types: “sleep bruxism” (nocturnal) and “awake bruxism” (diurnal). The American Academy of Sleeping Medicine recommends that these two types be separated because of different aetiologies and presumed risk factors [4]. Awake bruxism is defined as awareness of jaw clenching [6,7,8]. The prevalence of bruxism is around 10% and declines gradually with age [6,7,9], and the prevalence of awake bruxism (8–34%) [9,10,11,12,13,14,15] is higher than that of sleep bruxism (9.7–15.9%) [9,16].

Bruxism can seriously affect quality of life, and induce problems such as pain, temporomandibular disorders and failure of prosthetic treatments [8,17,18,19,20]. The large forces involved in bruxism can have detrimental effects on the components of the masticatory system [18]. A load of more than 20 g over periods of 2.5 s per clenching might be imposed on a tooth, exceeding normal functional stresses [18]. Such force can induce tooth movement and contribute to malocclusion [21].

In a previous cross-sectional study, the prevalence of malocclusion (crowding) was significantly associated with awareness of clenching in university students [22]. Since malocclusion provides functional and aesthetic disturbances, and may lead to psychological stress [23], prevention or control are important for dental clinicians. Confirmation of causal relationships in a prospective cohort study is therefore necessary. Based on our previous study involving young adults [22], we hypothesized that clenching during the daytime as awake bruxism would be a risk factor for malocclusion. We configured the null hypothesis in which clenching does not induce malocclusion. The purpose of this prospective cohort study was to investigate the association between the onset of malocclusion and awareness of clenching during the daytime in young adults. We could clarify a part of association between clenching and malocclusion.

## 2. Materials and Methods

### 2.1. Study Population

The present study used a prospective cohort design. We estimated the sample size based on previous studies using SamplePower version 3.0 statistical software (IBM, Tokyo, Japan). For logistic regression analysis, this software computed power for a test of the null hypothesis in which the event rate in the two groups was identical. According to a previous study [22], the minimum sample size required in both positive and negative groups to detect significant differences in the awareness of clenching during the daytime was 55 with event rate (0.38 and 0.04), 80% power and a two-sided significance level of 5%. Assuming a follow-up rate of 26.2% [24], the planned minimum sample size was 209 participants. 

We obtained data from first-year students who had undergone both a general health and oral examination at the Health Service Center of Okayama University in April 2013 (baseline). The inclusion criteria at baseline were Japanese students 18 or 19 years of age who did not show malocclusion, did not have experience receiving orthodontic treatment, and provided complete data in their questionnaires. Before graduation, students volunteered to receive both the general health and oral examination in April 2016 (follow-up). We excluded students who did not undergo the oral examination at follow-up, began orthodontic treatment during a residence, or provided incomplete data in their questionnaires at follow-up.

### 2.2. Ethical Procedures and Informed Consent

All study protocols were approved by the ethics committees of Okayama University Graduate School of Medicine, Dentistry and Pharmaceutical Sciences and Okayama University Hospital (no. 1512-018). All targeted participants gave their informed written consent for study participation. STROBE guidelines were followed (Table A1).

### 2.3. Self-Questionnaires

At baseline (2013), students answered questions concerning their name, age, sex, general condition, experience of orthodontic treatment, awareness of bruxism, and oral habits at baseline [22]. According to a previous study [22], we asked students to identify their awareness of bruxism as follows: during the past 3 months, (i) “Has anyone heard you grinding your teeth at night?”; (ii) “Are you ever aware of grinding your teeth during the daytime?”; and (iii) “Are you ever aware of clenching your teeth during the daytime?” [8,22,25,26,27]. Each question was answered by selecting a frequency (frequently, sometimes, rarely, or never). We combined “rarely” and “never” responses into a single category of negative awareness, and the remaining two responses into a single category of positive awareness [22]. The validity and reliability of the questionnaire have been confirmed as useful for evaluating bruxism [8,25,26,27]. For oral habits, “yes/no” answers were given by participants as follows: biting fingernails/pens/pencils, biting mucosa of the cheeks/lips, and gum chewing [22,28,29,30]. At follow-up in 2016, students provided their history of orthodontic treatment during the 3 year study period, non-nutritive sucking [22], habitual mouth breathing [31], early loss of primary teeth [32], and parents’ history of malocclusion in a “yes/no” format.

### 2.4. Assessment of Malocclusion

Five dentists (D.E., K.K., M.Y-T., S.M., and T.A.) examined malocclusion in the participants during the oral examinations. We used a modified version of the Index of Orthodontic Treatment Need (IOTN) that does not define a definite aesthetic need for treatment (Aesthetic Component grades 8, 9 and 10) to assess malocclusion (Table 1) [23]. Our modified version of the IOTN and the original modified version [33] are useful for screening malocclusion by non-orthodontists in oral health surveys [22]. The dental health component of the modified IOTN consists of a two-grade scale (0 = no definite need for orthodontic treatment; i.e. we defined it as a normal occlusion [normal occlusion group]; 1 = definite need for orthodontic treatment; i.e. we defined it as a malocclusion [malocclusion group]). The type of malocclusion (missing teeth, overjet, crossbite, crowding, or overbite) was recorded using the community periodontal index (CPI) probe (YDM, Tokyo, Japan) in accordance with a previous study [22]. When one of them was positive at least, we defined that the participant had malocclusion. All dentists were trained and calibrated to use the modified IOTN. For this, an orthodontist acted as the gold standard. The kappa value was > 0.8.

### 2.5. Assessment of Body Mass Index (BMI)

In the general health examination at baseline and follow-up, public health nurses at the university measured the participants’ height and body weight using a Tanita body fat analyser (BF-220; Tanita Co., Tokyo, Japan). Since BMI may be related to jaw growth, BMI was computed as weight in kilograms divided by height in meters squared. For this analysis, categories of BMI were calculated based on the accepted cut-off values for underweight (BMI < 18.5 kg/m^2^), normal weight (BMI 18.5–24.9 kg/m^2^), and overweight (BMI ≥ 25.0 kg/m^2^) [34].

### 2.6. Statistical Analyses

We used SPSS version 20 (IBM, Tokyo, Japan) for statistical analyses. *P* values < 0.05 were considered to indicate significant associations. The McNemar–Bowker or paired *t*-test was used to investigate significant differences between baseline and follow-up. The chi-square test was used to determine significant differences between the normal occlusion and malocclusion groups, whereas in cases of awareness of bruxism at baseline and oral habits at baseline, the chi-square test with Bonferroni correction to control the false discovery rate (*P* < 0.05/3) was used [35]. Since the majority (approximately 83%) of malocclusions involved crowding, we also investigated associations between crowding and other parameters.

Odds ratios (ORs) and 95% Confidence Intervals (CIs) were calculated using a series of logistic regression models. The onset of malocclusion or crowding was used as the dependent variable. Based on binary analyses and previous studies [22], BMI category, clenching during the daytime, and sex were added as independent variables in multiple logistic regression models as items associated with outcome. We assessed model fit using the Hosmer–Lemeshow goodness-of-fit test for logistic regression.

## 3. Results

### 3.1. Study Population

**Figure 1:**
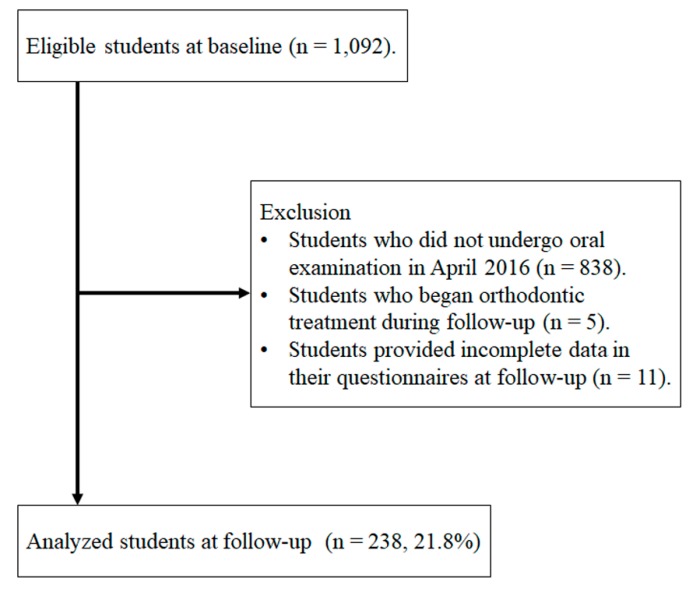
Flow chart showing protocols for selecting analysed students from among first-year students who did not meet the exclusion criteria (malocclusion conditions, age and orthodontic treatment).

Figure 1 shows a flow chart of participants in this three-year cohort study from baseline to follow-up. We selected 1,092 students who matched the study criteria at baseline. At the follow-up, 838 students had not undergone an oral examination and 16 met the exclusion criteria (five students had received orthodontic treatment and 11 had provided incomplete data). Finally, 238 students were analysed (normal occlusion vs. malocclusion). The follow-up rate was 21.8% (238/1092). Furthermore, we performed subgroup analysis to focus on crowding. We excluded 22 students who had other types of malocclusion (overjet, overbite, crossbite, and missing teeth). Finally, 216 students were analysed (normal occlusion vs. crowding).

### 3.2. Changes in Parameters from Baseline to Follow-Up

The incidences of malocclusion and crowding were 53.8% and 44.5%, respectively (Table 2). No significant difference in BMI distribution was seen between baseline and follow-up (McNemar–Bowker tests; *P* > 0.05). On the other hand, mean height and weight differed significantly between stages (paired *t*-test; *P* < 0.05; 95%CI of height, 0.15–0.33; 95%CI of weight, 0.13–1.15).

### 3.3. Association between Malocclusion/Crowding and Other Parameters

Among the parameters examined, a significant difference in BMI distribution was seen between the normal occlusion and malocclusion groups (chi-square test; *P* = 0.04) (Table 3). Significant difference in BMI distribution were seen during the daytime between the normal occlusion and crowding groups (chi-square test; *P* = 0.02) (Table 4).

Logistic regression analysis showed the risk of malocclusion was significantly related to underweight (BMI < 18.5 kg/m^2^; multiple logistic regression analysis; *P* = 0.03; 95%CI, 1.11–4.92) (Table 5). However, no significant association was identified between malocclusion and awareness of clenching during the daytime (multiple logistic regression analysis; *P* > 0.05; 95%CI, 0.91–9.88). The Hosmer–Lemeshow test found acceptable model fit, with a chi-square statistic of 0.78 (*P* = 0.68). 

On logistic regression analysis, risk of crowding correlated significantly with underweight (BMI < 18.5 kg/m^2^) and awareness of clenching during the daytime (multiple logistic regression analysis; *P* < 0.05) (Table 5). The Hosmer–Lemeshow test found acceptable model fit, with a chi-square statistic of 0.88 (*P* = 0.83).

## 4. Discussion

A previous cross-sectional study showed a significant association between prevalence of malocclusion (crowding) and awareness of clenching in Japanese university students [22]. To the best of our knowledge, the present study is the first prospective cohort study to investigate whether awareness of clenching during the daytime is a risk factor for malocclusion in young adults. In this study, the results showed that awareness of clenching during the daytime was associated with the onset of crowding (adjusted OR, 3.63; 95% CI, 1.08–12.17). These findings may support our hypothesis that awareness of clenching during the daytime is a risk factor for crowding.

Previous studies have suggested that clenching forces contribute to tooth movement [21,36,37]. This force can induce tooth movement and contribute to malocclusion [21]. The mean clenching force is 720 N (162 lb) with a range of 244–1243 N (55–280 lb) [36]. The bite force needed to contribute to displacement is approximately 100 N [18,38]. Because the force of clenching is higher than the threshold of tooth displacement, clenching during the daytime may represent a risk factor for crowding through tooth movement.

Underweight (BMI < 18.5 kg/m^2^) was associated with the onset of crowding. Underweight is related to skeletal maturation [39], and delayed maturation might affect the onset of malocclusion [40]. Kataoka et al. [22] showed a significant association between underweight and the prevalence of malocclusion in a cross-sectional study. In the present study, mean height in students with normal weight (18.5 ≤ BMI < 25 kg/m^2^) at baseline increased significantly during the 3-year study period (164.6 cm [standard deviation (SD), 8.3 cm] at baseline vs. 164.9 cm [SD, 8.5 cm] at follow-up; paired *t*-test, [SD of the difference, 0.7 cm], *P* < 0.001). However, mean height in students with underweight (BMI < 18.5 kg/m^2^) was not significantly increased (164.0 cm [SD, 8.8 cm] at baseline vs. 164.2 cm [SD, 9.2 cm] at follow-up; paired *t*-test, [SD of the difference, 0.8 cm], *P* > 0.05). These results suggest that underweight students have less skeletal maturation or less maxillary and/or mandibular growth. Taken together, underweight could affect the onset of malocclusion through reduced skeletal maturation.

Oral habits, including biting fingernails/pens/pencils, biting the mucosa of the cheeks/lips, and gum chewing [28,29,30], were not significantly associated with the onset of malocclusion (chi-square test; *P* >0.05). Previous studies have shown that bad oral habits are related to the prevalence of malocclusion [41,42]. Some inconsistencies are apparent between our study and previous studies. The reasons for this are unclear, but may involve differences in age (young adults vs. children) and dentition (permanent vs. primary).

The results of the present study might be clinically relevant. Treatment for awake bruxism is based on behaviour modification and habit reversal. Behaviour modification has the potential to stop or reduce awake bruxism [2,43]. Treatment for awake bruxism could therefore prevent the onset of malocclusion. When clinicians encounter younger patients who are aware of clenching during the daytime, increased efforts may be needed to prevent malocclusion. The present work was only an observational study. Further studies are therefore needed to clarify whether clinical interventions can help prevent the onset of malocclusion.

The prevalence of malocclusion was higher than that of the previous studies. In the present study, the rate of onset of malocclusion was 53.8% (Table 2). In previous research using IOTN, the prevalence of malocclusion varied widely among subjects (21–44.9%) [23,44,45,46]. As participant age, country, sample size, and study design differed between the present and other investigations, caution is warranted in regard to the generalizability of the results. 

The participants were not considered an unusual sample based on the two aspects as below. The prevalence of awake bruxism was 8.0% in the present work, which is within the 8–34% range reported in previous studies [9,10,11,12,13,14,15]. Moreover, the distributions of underweight, normal weight, and overweight based on BMI classifications established by the World Health Organization were 17.2%, 75.6% and 7.1%, respectively. As a reference, the 2013 Japan National Health and Nutrition Survey showed distributions of underweight, normal weight, and overweight of 22.3%, 70.2%, and 7.4%, respectively (age range, 15–19 years) [47]. The distribution of BMI categories in the present study did not differ significantly from that in the Japanese national survey (chi-square test; *P* > 0.05). 

No relationship was observed between sex and the onset of malocclusion. Previous studies have also reported that sex was not significantly associated with the prevalence of malocclusion based on the IOTN [48,49,50]. Those studies support our results.

Several limitations of the present study must be considered when interpreting the results. First, these findings to young adults in general. Second, we did not investigate bite force or clenching force; these are difficult to measure in general oral examinations because they require special instruments [51]. Third, there may have been a selection bias, given the low follow-up rate. In the present study, analysed students (n = 238) comprised 21.8% of all eligible students (n = 1,092). However, no significant differences were seen in the ratios of bruxism, oral habits, or BMI between the analysed and non-analysed students (238 vs. 854 students, chi-square test; *P* > 0.05), with the exception of sex (chi-square test; *P* < 0.05). Any effects of a selection bias would have therefore been negligible. Forth, we could not investigate malocclusion in the participants’ parents. We have to pay attention to deal with the data of parents’ malocclusion based on the questionnaire because of bias. Finally, we did not investigate tongue thrust, which might affect malocclusion, based on a recent case report [52].

## 5. Conclusions

In conclusion, in this prospective cohort study, awareness of clenching during the daytime and underweight were found to be related to the onset of malocclusion (crowding) among university students. These findings suggest that clinicians may need to apply increased efforts to prevent malocclusion in younger patients who are aware of clenching during the daytime.

## Figures and Tables

**Table 1:** Modified version of the Index of Orthodontic Treatment Need [22,33].

Missing teeth	Hypodontia requiring pre-restorative orthodontics or orthodontic space closure to obviate the need of a prosthesis.
	Impeded eruption of teeth, presence of supernumerary teeth, and retained deciduous teeth.
Overjet	Increased overjet greater than 6 mm.
	Reverse overjet greater than 3.5 mm with no masticatory or speech difficulties.
	Reverse overjet greater than 1 mm but less than 3.5 mm with recorded masticatory and speech difficulties.
Crossbite	Anterior or posterior crossbites with greater than 2 mm discrepancy between retruded contact position and intercuspal position.
Displacement of contact points (crowding)	Contact point displacements greater than 4 mm.
Overbite	Lateral or anterior open bites greater than 4 mm.
	Deep overbite with gingival or palatal trauma.

**Table 2:** Changes in parameters from baseline to re-examination among Okayama University students, Japan, 2013–2016.

Parameters	Total (n = 238)		95%CI	*P* Value
	Baseline	Follow-Up Period		
	n (%)	n (%)		
	Mean ± SD	Mean ± SD		
Malocclusion	0 (0.0)	128 (53.8)		
Crowding	0 (0.0)	106 (44.5)		
BMI (kg/m^2^)				
Normal range (18.5 ≤ BMI < 25)	180 (75.6)	187 (78.6)		0.389 ^1^
Underweight (<18.5)	41 (17.2)	35 (14.7)		
Overweight (≥25)	17 (7.1)	16 (6.7)		
Height (cm)	164.8 ± 8.3	165.0 ± 8.5	0.15–0.33	<0.001 ^2^
Weight (kg)	56.4 ± 10.0	57.1 ± 9.7	0.13–1.15	0.015 ^2^

SD, standard deviation; BMI, body mass index. ^1^ McNemar–Bowker test. ^2^ Two-sided *P* values were based on a paired *t*-test.

**Table 3:** Association between onset of malocclusion and other parameters among Okayama University students, Japan, 2013–2016.

Parameter	Normal Occlusion n = 110	Malocclusion n = 128	*P* Value ^1^
	n (%)	n (%)	
Sex			
Male	56 (50.9)	70 (54.7)	0.560
Awareness of bruxism at baseline			
Grinding during daytime			
Yes	2 (1.8)	4 (3.1)	0.689
Clenching during daytime			
Yes	4 (3.6)	13 (10.2)	0.052
Sleep bruxism			
Yes	10 (9.1)	7 (5.5)	0.279
Oral habits at baseline			
Gum chewing			
Yes	12 (10.9)	10 (7.8)	0.411
Biting fingernail/pens/pencils			
Yes	10 (9.1)	11 (8.6)	0.893
Biting mucosa of cheeks/lips			
Yes	22 (20.0)	26 (20.3)	0.952
Early loss of primary teeth			
Yes	9 (8.2)	4 (3.1)	0.087
Presence of malocclusion in parents			
Yes	5 (4.5)	14 (10.9)	0.070
BMI at baseline (kg/m^2^)			
Normal range (18.5 ≤ BMI < 25)	91 (82.7)	89 (69.5)	0.043
Underweight (<18.5)	12 (10.9)	29 (22.7)	
Overweight (≥25)	7 (6.4)	10 (7.8)	

BMI, body mass index. ^1^ Two-sided *P* values were based on the chi-square tests.

**Table 4:** Association between onset of crowding and other parameters among Okayama University students, Japan, 2013–2016.

Parameter	Normal Occlusion n = 110	Crowding n = 106	*P* Value ^1^
	n (%)	n (%)	
Sex			
Male	56 (50.9)	59 (55.7)	0.484
Awareness of bruxism at baseline			
Grinding during daytime			
Yes	2 (1.8)	4 (3.8)	0.439
Clenching during daytime			
Yes	4 (3.6)	12 (11.3)	0.031
Sleep bruxism			
Yes	10 (9.1)	6 (5.7)	0.336
Oral habits at baseline			
Gum chewing			
Yes	12 (10.9)	7 (6.6)	0.264
Biting fingernail/pens/pencils			
Yes	10 (9.1)	10 (9.4)	0.931
Biting mucosa of cheeks/lips			
Yes	22 (20.0)	21 (19.8)	0.972
Early loss of primary teeth			
Yes	9 (8.2)	4 (3.8)	0.173
Presence of malocclusion in parents			
Yes	5 (4.5)	11 (10.4)	0.102
BMI at baseline (kg/m^2^)			
Normal range (18.5 ≤ BMI < 25)	91 (82.7)	71 (67.0)	0.020
Underweight (<18.5)	12 (10.9)	26 (24.5)	
Overweight (≥25)	7 (6.4)	9 (8.5)	

BMI, body mass index. ^1^ Two-sided *P* values were based on the chi-square test.

**Table 5:** Adjusted odds ratios and 95% confidence intervals for the onset of malocclusion or crowding among Okayama University students, Japan, 2013–2016.

Variables	Malocclusion			Crowding		
	OR	95%CI	*P* Value ^1^	OR	95%CI	*P* Value ^1^
Sex						
Female	1.00	Ref		1.00	Ref	
Male	1.34	0.79–2.29	0.279	1.45	0.82–2.55	0.183
Clenching during daytime						
No	1.00	Ref		1.00	Ref	
Yes	3.00	0.91–9.88	0.070	3.63	1.08–12.17	0.037
BMI at baseline (kg/m^2^)						
Normal range (18.5 ≤ BMI < 25)	1.00	Ref		1.00	Ref	
Underweight (<18.5)	2.34	1.11–4.92	0.025	2.52	1.25–5.76	0.011
Overweight (≥25)	1.41	0.51–3.91	0.505	1.67	0.57–4.58	0.373

CI, confidence interval; OR, odds ratio; BMI, body mass index. ^1^ Multiple logistic regression model adjusted for sex, BMI and clenching during daytime.

## Appendix A

**Table A1:** STROBE Statement.

	Item No.	Recommendation	Page No.
**Title and abstract**	1	(*a*) Indicate the study’s design with a commonly used term in the title or the abstract	1
		(*b*) Provide in the abstract an informative and balanced summary of what was done and what was found	1
**Introduction**			
Background/rationale	2	Explain the scientific background and rationale for the investigation being reported	1-2
Objectives	3	State specific objectives, including any prespecified hypotheses	2
**Methods**			
Study design	4	Present key elements of study design early in the paper	2
Setting	5	Describe the setting, locations, and relevant dates, including periods of recruitment, exposure, follow-up, and data collection	2
Participants	6	(*a*) Give the eligibility criteria, and the sources and methods of selection of participants. Describe methods of follow-up	2
		(*b*) For matched studies, give matching criteria and number of exposed and unexposed	N/A
Variables	7	Clearly define all outcomes, exposures, predictors, potential confounders, and effect modifiers. Give diagnostic criteria, if applicable	2-3
Data sources/measurement	8 *	For each variable of interest, give sources of data and details of methods of assessment (measurement). Describe comparability of assessment methods if there is more than one group	2-3
Bias	9	Describe any efforts to address potential sources of bias	2
Study size	10	Explain how the study size was arrived at	2
Quantitative variables	11	Explain how quantitative variables were handled in the analyses. If applicable, describe which groupings were chosen and why	3
Statistical methods	12	(*a*) Describe all statistical methods, including those used to control for confounding	4
		(*b*) Describe any methods used to examine subgroups and interactions	4
		(*c*) Explain how missing data were addressed	2
		(*d*) If applicable, explain how loss to follow-up was addressed	2
		(*e*) Describe any sensitivity analyses	N/A
**Results**			
Participants	13 *	(a) Report numbers of individuals at each stage of study—eg numbers potentially eligible, examined for eligibility, confirmed eligible, included in the study, completing follow-up, and analysed	4
		(b) Give reasons for non-participation at each stage	4
		(c) Consider use of a flow diagram	Figure 1
Descriptive data	14 *	(a) Give characteristics of study participants (eg demographic, clinical, social) and information on exposures and potential confounders	5
		(b) Indicate number of participants with missing data for each variable of interest	4
		(c) Summarise follow-up time (eg, average and total amount)	4
Outcome data	15 *	Report numbers of outcome events or summary measures over time	5
Main results	16	(*a*) Give unadjusted estimates and, if applicable, confounder-adjusted estimates and their precision (eg, 95% confidence interval). Make clear which confounders were adjusted for and why they were included	5-6
		(*b*) Report category boundaries when continuous variables were categorized	5-7
		(*c*) If relevant, consider translating estimates of relative risk into absolute risk for a meaningful time period	N/A
Other analyses	17	Report other analyses done—eg analyses of subgroups and interactions, and sensitivity analyses	6-7
**Discussion**			
Key results	18	Summarise key results with reference to study objectives	7
Limitations	19	Discuss limitations of the study, taking into account sources of potential bias or imprecision. Discuss both direction and magnitude of any potential bias	8
Interpretation	20	Give a cautious overall interpretation of results considering objectives, limitations, multiplicity of analyses, results from similar studies, and other relevant evidence	8
Generalisability	21	Discuss the generalisability (external validity) of the study results	8
**Other information**			
Funding	22	Give the source of funding and the role of the funders for the present study and, if applicable, for the original study on which the present article is based	9

* Give information separately for exposed and unexposed groups.
